# Civilian mass exposure to hydrazine after an F-16 crash: a retrospective descriptive study

**DOI:** 10.1186/s12873-025-01373-y

**Published:** 2025-10-22

**Authors:** Asli Bahar Ucar, Hasan Demir, Haldun Akoglu

**Affiliations:** 1 Department of Emergency Medicine, Marmara University Pendik Training and Research Hospital, Istanbul, 34899 Türkiye; 2 Department of Emergency Medicine, Fatih Sultan Mehmet Training and Research Hospital, Istanbul, Türkiye; 3 Department of Emergency Medicine, Marmara University Faculty of Medicine, Istanbul, Türkiye

**Keywords:** Aircraft, Emergency medicine, Hydrazine, Inhalation exposure, Poisoning, Public health, Toxicology

## Abstract

**Background:**

Hydrazine is a volatile and highly toxic monopropellant used in the Emergency Power Unit (EPU) of F-16 aircraft. Although hydrazine is widely used in both military and industrial contexts, reports of its effects in non-occupational civilian populations are extremely limited. Acute civilian mass exposures are almost never systematically documented. On December 12, 2016, an F-16 crash in Diyarbakır, Türkiye, released hydrazine into an open-field setting, exposing unprotected civilians. This study aimed to characterize acute manifestations, evaluate associations with exposure duration and route, and identify operational implications.

**Methods:**

We conducted a retrospective descriptive study of 30 previously healthy male civilians exposed at the crash site. Clinical data, laboratory findings, ECGs, and imaging were retrieved from standardized emergency department records. Exposure duration—triangulated from patient interviews and operational logs—was analyzed as a continuous surrogate for dose, in line with Haber’s rule. Associations with symptom categories and exposure routes were assessed using nonparametric statistics, Fisher’s exact tests, and exploratory receiver operating characteristic (ROC) analysis.

**Results:**

Twenty patients (66.7%) developed symptoms, while ten remained asymptomatic. Symptom development correlated strongly with exposure duration (Spearman *r* = 0.61–0.68), with respiratory cases showing significantly longer exposures (median 45 vs. 14 min, *p* = 0.0017). Ophthalmologic findings (36.7%) were most frequent, while dermatologic (26.7%) and respiratory (13.3%) manifestations occurred exclusively in contact-exposed individuals (*p* < 0.001 and *p* = 0.012, respectively). Inhalation-only exposure primarily caused ocular irritation. No patient exposed ≤ 5 min developed symptoms, whereas all symptomatic individuals reported longer exposures; ROC analysis supported discriminatory potential (AUC > 0.80), though results remain exploratory. A 26-year-old first responder developed acute hypoxemic respiratory failure after 45–60 min, recovering with supportive care. No hepatic, renal, neurologic, or cardiovascular complications occurred, and all patients were discharged within 24 h.

**Conclusions:**

This study provides one of the most extensive civilian datasets on acute hydrazine exposure. Findings highlight a strong dose–time relationship and route-specific patterns, with exposures > 5 min associated with clinically significant toxicity. While thresholds remain hypothesis-generating, all patients recovered rapidly with supportive care. Operational lessons include the need for rapid evacuation, immediate decontamination, and strict use of personal protective equipment, offering practical insights for toxicology practice and disaster preparedness.

**Supplementary Information:**

The online version contains supplementary material available at 10.1186/s12873-025-01373-y.

## Introduction

Hydrazine (N₂H₄) is a colorless, flammable compound widely used in rocket propellants, emergency power units, industrial corrosion control, and numerous chemical processes. It is also present in pesticides, plastic foams, textile dyes, and tobacco smoke, and is used pharmaceutically in the production of cefazolin, isoniazid, allopurinol, and fluconazole [1–3].

Hydrazine exposure may occur by inhalation, ingestion, or dermal contact, with toxic effects on the lungs, liver, kidneys, nervous system, and mucous membranes. The liquid form is corrosive and may cause chemical burns or severe dermatitis, while vapor exposure can result in conjunctivitis, skin irritation, and respiratory injury, including pulmonary edema [1–4]. Ingestion can provoke seizures, CNS depression, and coma [1–5]. Despite extensive industrial and military use, acute hydrazine exposure in civilian populations is rarely documented, and systematic clinical descriptions remain scarce.

Globally, more than 100,000 metric tons of hydrazine produced annually still lead to occasional human exposures in both military and civilian contexts. The most comprehensive dataset to date is from Nguyen et al., who analyzed 20 years of poison center reports in the United States [5]. A fatal case with liver necrosis following inhalation was reported by Sotaniemi et al. [6].

Hydrazine is utilized as a propellant in the gas turbine of the Emergency Power Unit (EPU) of the F-16 Fighting Falcon, where it is stored in the form of H-70—a mixture comprising 70% hydrazine and 30% water. The EPU, located in the upper fuselage just above and forward of the right wing, is critical for providing emergency hydraulic and electrical power to the aircraft’s flight control systems. It contains approximately 25.4 kg (56 lb; 6.8 gal) of H-70 and is equipped with a dedicated leak detection system [2, 7]. This system employs a pellet containing a hydrazine-sensitive chemical indicator, which undergoes a distinct colorimetric change from yellow to deep purple or black upon exposure, thereby providing an unambiguous and rapid visual confirmation of leakage [2, 7, 8]. Quantitative measurement of hydrazine concentration is not routinely feasible, because even highly sensitive analytical techniques such as high-performance liquid chromatography (HPLC) require detection limits in the range of 0.0012 ppm (0.16 ng per sample), while simpler colorimetric screening methods have detection limits of approximately 0.005 ppm [9].

On 12 December 2016, in Diyarbakir, Türkiye, an F-16 fighter jet crashed during the landing phase of a training flight following a technical malfunction. The pilot ejected safely and remained unharmed, with no exposure to hydrazine. However, hydrazine leaked from the aircraft’s emergency power unit (EPU) at the crash site. This study documents the clinical symptoms of 30 civilians who arrived at the scene to rescue the pilot before rescue teams arrived and who did not have protective equipment.

This study aimed to describe the acute clinical manifestations of hydrazine exposure in civilians following an F-16 crash and to evaluate the relationship between exposure duration and symptom development, along with exposure route, laboratory findings, and complications, to provide a broader clinical perspective.

## Methods

### Study design

This was a retrospective descriptive study conducted to evaluate the acute clinical effects of hydrazine exposure in a civilian population. The incident occurred on December 12, 2016, when an F-16 Fighting Falcon crashed in Diyarbakir, Türkiye, releasing H-70 fuel. The study included 30 previously healthy male civilians who were present at the crash site and were not wearing any protective equipment. Approval for the evaluation of patient data was granted by the Diyarbakır Selahaddin Eyyubi Chief Medical Officer’s Office. The study was also approved by the Ethics Committee of the Fatih Sultan Mehmet Training and Research Hospital, Istanbul (Approval date: May 25, 2023; Protocol No: FSMEAH-KAEK 2023/75) and conducted in accordance with the Declaration of Helsinki.

### Study population and data collection

All 30 patients who presented to the emergency department were civilians; none were military personnel or had pre-existing medical conditions. Demographic characteristics (age, medical history), vital signs, Glasgow Coma Scale (GCS) scores, laboratory test results, electrocardiograms (ECGs), and radiologic imaging findings were retrospectively retrieved from the hospital information system, admission records, and nursing documentation. Patient data were abstracted independently by two physicians using a predefined template under institutional authorization, and any discrepancies were resolved by consensus. The cases were blinded to the abstracting physicians to minimize potential bias.

Laboratory investigations—including blood gas analysis, complete blood count, serum chemistry, and ECG—were performed in all patients, including asymptomatic individuals, as part of the standardized Türkiye Ministry of Health Chemical, Biological, Radiological, and Nuclear (CBRN) protocol. Imaging studies (chest X-ray or computed tomography) were obtained based on clinical indication.

Exposure duration was estimated by triangulating patient interviews with time-stamped emergency call logs, scene arrival records, and notations from on-site military responders. In the absence of quantitative hydrazine concentration measurements at the crash site, exposure time was pragmatically used as a surrogate of dose, consistent with Haber’s rule (C×t = k) [10]. No quantitative air sampling was performed, as validated occupational methods (e.g., OSHA Method 20) require pump-based collection, derivatization, and laboratory analysis, which are not feasible during acute incidents [9, 11]. Details of statistical analyses are provided in the Statistical Analysis section.

The route of exposure (inhalation, contact, or both) was documented for each patient. Symptoms were classified into ophthalmologic (e.g., conjunctival irritation, visual blurring), dermatologic (e.g., erythema, rash, burning), respiratory (e.g., cough, dyspnea, wheezing), and gastrointestinal (e.g., nausea, vomiting, diarrhea) categories. Complications such as bronchospasm or pulmonary infiltrates were recorded only if documented by physicians and/or confirmed by imaging. A dedicated ward was arranged, and all patients were admitted for observation until their symptoms resolved.

At discharge, all patients received standardized written and verbal return precautions outlining emergency symptoms that should prompt immediate medical evaluation. A telephone follow-up was conducted at 7 days to assess for new or persistent symptoms, and no delayed symptoms were reported.

### Study outcomes

The primary outcome was the association between hydrazine exposure duration and the development of acute clinical symptoms, categorized as ophthalmologic, dermatologic, respiratory, or gastrointestinal.

Secondary outcomes included:

1. Assessment of symptom distribution according to the suspected route of exposure (contact, inhalation, or both);
2. Characterization of clinically confirmed complications;
3. Exploratory identification of an exposure-duration threshold to distinguish symptomatic from asymptomatic individuals.

### Statistical analysis

Continuous variables were summarized as mean ± SD or median (IQR) according to Shapiro–Wilk normality testing. Categorical variables were presented as counts and percentages. Exposure time was analyzed as a continuous predictor: associations with symptom categories were assessed using Spearman’s rank correlation, independent-samples t tests (with Welch’s correction when variances were unequal by Levene’s test), or Mann–Whitney U tests, as appropriate. Fisher’s exact test was used for categorical comparisons with small cell counts.

An exploratory receiver operating characteristic (ROC) analysis was performed to evaluate whether an exposure-duration threshold could distinguish symptomatic from asymptomatic individuals. The exploratory threshold was identified using Youden’s J statistic; AUC point estimates and bootstrap 95% confidence intervals (1,000 resamples) are reported in Supplementary Table S1. Given the small sample size, midpoint-imputed exposure durations, and absence of quantitative concentration measurements, this analysis is considered hypothesis-generating and dataset-specific.

Exposure minutes were approximated from reported duration bins using midpoints (< 5 = 2.5; 5–15 = 10; 15–30 = 22.5; 30–45 = 37.5; 45–60 = 52.5 min). Analyses were conducted using SPSS Statistics Version 26.0 (IBM Corp., Armonk, NY) and Jamovi Version 2.3. Two-sided *p* < 0.05 was considered statistically significant. Odds ratios with 95% confidence intervals were computed; when zero cells occurred, the Haldane–Anscombe correction was applied. Rank-biserial correlations with bootstrap 95% CIs (3,000 resamples) were reported for Mann–Whitney U tests. Where applicable, effect sizes with 95% confidence intervals are provided in Supplementary Table S2.

## Results

Initial presentations began within the first hour after the crash; all 30 individuals arrived at the emergency department within the first few hours. All patients were male civilians (mean age 30.8 ± 8.4 years) with no prior military service or pre-existing medical conditions. Twenty patients (66.7%) developed symptoms, while ten remained asymptomatic but presented out of concern for potential delayed effects. In all 10 asymptomatic individuals, laboratory investigations (arterial blood gas, troponin, serum biochemistry, complete blood count, and ECG) were performed as part of CBRN protocol to anticipate potential effects; no abnormalities were detected, and no pathology developed during 7- day follow-up. The 2019 document has since been updated online, but our practice at the time reflected the earlier version available in 2016, whose recommendations for chemical agent management were consistent with the current guideline [12] .

All patients underwent management per the CBRN protocol: removal of clothing, thorough soap-and-water decontamination, and provision of clean garments. Ocular irrigation was performed as needed, and all patients were admitted for observation until resolution [12].

Among symptomatic patients (*n* = 20, 66.7%), ophthalmologic findings were most common (*n* = 11, 36.7%), followed by dermatologic (*n* = 8, 26.7%) and respiratory (*n* = 4, 13.3%) complaints; gastrointestinal symptoms were observed in only one patient and appeared only with ocular irritation. Dermatologic manifestations included hyperemic rash alone in five patients, rash with dyspnea in two, and rash with conjunctivitis in one. Four patients experienced complaints affecting more than one system (Table 1). Dermatologic and respiratory findings occurred exclusively in patients with direct contact (8/11 and 4/11, respectively), while ophthalmologic findings predominated in the inhalation-only group (9/20).

**Table 1:** Distribution of symptom combinations among symptomatic patients (*n* = 20)

Symptom combination	*n*	% of symptomatic patients*	Mean exposure time (min) ± SD
Only Ophthalmologic	9	45.0%	20.0 ± 8.9
Only Dermatologic	5	25.0%	22.5 ± 7.5
Only Respiratory	2	10.0%	45.0 ± 7.1
Dermatologic + Respiratory	2	10.0%	42.5 ± 10.6
Dermatologic + Ophthalmologic	1	5.0%	30.0 ± –
Ophthalmologic + Gastrointestinal	1	5.0%	37.5 ± –
**Total**	20	100%	–

*Percentages are calculated over 20 symptomatic patients; asymptomatic individuals (*n* = 10) not shown. Mean exposure times are provided; SD not applicable for single-patient categories. Descriptive only; no formal statistical testing performed

Dermatologic findings occurred exclusively in the contact group (0/20 vs. 8/11; Fisher’s exact *p* = 2.8 × 10⁻⁵; OR 94.7, 95% CI 4.39–2041.9). Respiratory findings were also confined to the contact group (0/20 vs. 4/11; Fisher’s exact *p* = 0.012; OR 23.4, 95% CI 1.12–489.5). Ophthalmologic complaints were more frequent after inhalation-only exposure (9/20 vs. 2/11; Fisher’s exact *p* = 0.14; OR 0.247, 95% CI 0.042–1.460). Detailed effect size estimates with 95% confidence intervals are provided in Supplementary Table S2. Fisher’s exact testing confirmed significant associations for dermatologic (*p* < 0.001) and respiratory (*p* = 0.012) symptoms with contact exposure, but not for ophthalmologic symptoms (*p* = 0.14) (Table 2).

**Table 2:** Symptoms by exposure route with mean exposure time (minutes)

Symptom category	Inhalation only *n* (mean ± SD)	Contact & Inhalation *n* (mean ± SD)	Contact only *n* (mean ± SD)	Total min (overall mean ± SD)	*p*-value†
Ophthalmologic	9 (20.0 ± 8.9)	2 (23.8 ± 19.4)	–	11 (20.7 ± 10.2)	0.129
Dermatologic	0 (–)	8 (23.1 ± 10.4)	8 (23.1 ± 10.4)	8 (23.1 ± 10.4)	0.093
Respiratory	0 (–)	4 (45.0 ± 8.7)	4 (45.0 ± 8.7)	4 (45.0 ± 8.7)	**0.0017**‡
Gastrointestinal	0 (–)	1 (37.5)	1 (37.5)	1 (37.5)	0.208

† Two-sided Mann–Whitney U test comparing exposure minutes in those with vs. without the listed symptom; rank-biserial effect sizes with 95% CIs are reported in Supplementary Table S2

‡ Statistically significant (*p* < 0.05)

§ Fisher’s exact tests for symptom presence by route: Dermatologic 0/20 vs. 8/11 (*p* = 2.8 × 10⁻⁵; OR 94.7, 95% CI 4.39–2041.9); Respiratory 0/20 vs. 4/11 (*p* = 0.012; OR 23.4, 95% CI 1.12–489.5); Ophthalmologic 9/20 vs. 2/11 (*p* = 0.14; OR 0.247, 95% CI 0.042–1.460)

Exposure duration correlated strongly with the presence of ophthalmologic, dermatologic, and respiratory symptoms (Spearman *r* = 0.61, 0.63, and 0.68, respectively). Patients with respiratory symptoms had the longest exposure (45.0 ± 8.7 min), compared with 13.9 min in those without (Mann–Whitney U *p* = 0.0017; rank-biserial *r* = 0.962, 95% CI 0.885–1.000). No patient exposed ≤ 5 min developed symptoms, whereas all symptomatic individuals had > 5 min of exposure.

Exploratory ROC analysis indicated that in this dataset, symptom development was absent in individuals exposed ≤ 5 min, whereas all symptomatic cases had longer exposures. This apparent separation should be interpreted as a dataset-specific observation rather than a generalizable threshold, given the small sample size and the lack of quantitative exposure data (Supplementary Table S1).

A 26-year-old male first responder developed hydrazine inhalation–associated acute hypoxemic respiratory failure according to **ESICM guidelines** [13], after remaining near the F-16 crash site for approximately 45–60 min. On arrival, his oxygen saturation (SpO₂) was 84% on room air, respiratory rate 22 breaths/min, and blood pressure 146/72 mmHg. An initial arterial blood gas with co-oximetry obtained on room air showed a partial pressure of oxygen (PaO₂) of 70 mmHg, an arterial oxygen saturation (SaO₂) of 88%, and methemoglobin of 2.3% (reference 0–1.5%), with otherwise normal acid–base status and unremarkable blood gas parameters. Additional laboratory testing revealed hypochloremia (chloride 91 mmol/L) and leukocytosis (21 × 10^9/L). After initiation of supplemental oxygen via simple face mask at 6 L/min, SpO₂ was maintained at ≥ 94%. Cardiac evaluation (ECG and troponin) was unremarkable. Chest computed tomography demonstrated bilateral alveolar–interstitial infiltrates without cardiomegaly. The absence of peripheral edema, normal cardiac biomarkers, and the cardiology assessment supported non-cardiogenic pulmonary edema with acute hypoxemic respiratory failure attributable to hydrazine inhalation. As positive-pressure ventilation was not available at the hospital, the patient was treated for bronchospasm with corticosteroids (1 mg/kg methylprednisolone sodium succinate intravenously) and inhaled bronchodilators (salbutamol 2.5 mg, administered twice over 15 min via nebulization). In addition to oxygen support, he received 40 mg intravenous furosemide followed by a continuous infusion at 10 mg/h for 12 h, together with supportive oxygen therapy. Total urine output was ~ 3,000 mL; oxygen was weaned as oxygenation improved, and he was discharged in stable condition within 24 h. Methylene blue was not administered because the methemoglobin value was well below common treatment thresholds.

Two patients, including the one with hypoxemic respiratory failure, exhibited mild methemoglobinemia ( 2.3% and 2.2%) without systemic effects; methylene blue was withheld, and levels normalized within one hour. No patients developed seizures, hepatic, renal, or cardiac complications.

Abnormal laboratory markers—including methemoglobinemia > 1.5%, hypochloremia < 98 mmol/L, or leukocytosis > 10 × 10⁹/L—did not differ significantly by exposure duration. No patients demonstrated hepatic enzyme elevation, renal dysfunction, or ECG abnormalities. All were discharged within 24 h, and 7-day follow-up confirmed absence of delayed symptoms.

## Discussion

This study provides, to the best of our knowledge, one of the most comprehensive civilian datasets describing acute hydrazine exposure in an open-field setting. Unlike prior case-based reports or poison center reviews, our study population of 30 consecutively evaluated individuals allows for systematic clinical and statistical characterization of exposure-related manifestations. The principal finding was a clear time-dependent relationship: longer exposure was strongly associated with acute hypoxemic respiratory failure and other respiratory complaints, while ophthalmologic and dermatologic symptoms followed the same direction but did not reach statistical significance in this modest sample size. Because environmental concentrations were not measured, exposure duration was used as a pragmatic surrogate for dose—a field-adapted approach that provides unique insights despite its limitations.

In this study, no patient exposed for ≤ 5 min developed symptoms. While this separation was statistically apparent, it should be interpreted as hypothesis-generating and dataset-specific, pending validation in larger series with quantitative exposure data.

The clinical spectrum observed—ophthalmologic symptoms most frequent, followed by dermatologic and respiratory involvement—is in line with the mucocutaneous and pulmonary irritant properties of hydrazine described in toxicological literature. Our data also underscore a route-specific contribution: dermatologic symptoms occurred exclusively among individuals with physical contact, whereas inhalation-only exposures primarily caused ocular irritation. Gastrointestinal complaints were rare and occurred only in combination with other symptom categories, suggesting they are secondary rather than primary features of acute exposure.

Our findings align with prior reports. Nguyen et al. [5] analyzed poison center data (2000–2020) with 135 reported cases of acute hydrazine exposure, noting 57% were asymptomatic. He noted that acute hydrazine inhalation typically results in mucosal and respiratory irritation but seldom causes systemic hepatic or neurologic injury—consistent with our study, in which no hepatotoxic or neurologic abnormalities were detected and 33.3% of patients remained asymptomatic.

In contrast to our findings, Sotaniemi and colleagues have reported that chronic or high-concentration occupational exposures are associated with serious systemic consequences, including fatalities [6]. The absence of such findings here reinforces that a single, open-air exposure of short-to-moderate duration is less likely to cause multiorgan toxicity.

Frierson et al. reported two male patients aged 36 and 44 who were exposed to hydrazine vapor and developed pulmonary edema [3, 14]. Similarly, in our cases, the first responder, aged 26 years, developed non-cardiogenic pulmonary edema with acute hypoxemic respiratory failure, confirmed radiographically and by clinical criteria, but recovered rapidly with supportive therapy. Contemporary guidance for inhalational lung injury and acute hypoxemic respiratory failure emphasizes oxygen supplementation, noninvasive ventilation when indicated, and lung-protective strategies if intubation becomes necessary [13, 15, 16]. Although intravenous furosemide was administered in this case and symptomatic improvement was observed post diuresis, loop diuretics are not accepted as standard treatment for non-cardiogenic pulmonary edema [13]. Therefore, no causal inference should be drawn, and our report should not be interpreted as evidence in favor of diuretics in this context.

The role of pyridoxine in hydrazine toxicity remains unclear beyond its role in improving neurological symptoms. While case reports and biochemical rationale support its use in neurologic presentations (based on inhibition of pyridoxine to PLP conversion) [17, 18], it was not administered here, as none of the patients exhibited neurologic manifestations. No seizures, neuropathy, or persistent CNS findings—typically described in ingestion cases [16–18]—were observed, underscoring the importance of route and exposure duration in determining the clinical phenotype.

Interestingly, although several patients reported direct physical contact with wreckage debris, none developed cutaneous chemical burns, in contrast to industrial reports of severe dermal injury under pressurized or prolonged exposures [5, 19]. Cardiovascular abnormalities were likewise absent, consistent with limited evidence suggesting that acute cardiac involvement is rare in short-duration exposures [3, 5]. Collectively, these observations highlight operational priorities in similar future incidents: rapid evacuation from the exposure zone, immediate soap-and-water decontamination, protective equipment for responders, and triage protocols that incorporate exposure duration and route as key determinants of clinical risk. These findings underscore that even in open-air releases, minimizing exposure time remains critical to reducing clinical toxicity.

Although psychological stress and anxiety are relevant considerations in mass-casualty incidents, no acute stress reactions or panic were documented in our study population. Future studies should systematically evaluate psychological outcomes in addition to physical effects.

### Limitations

Hydrazine release was qualitatively confirmed by the Emergency Power Unit (EPU) leak-indicator pellet (color change). No quantitative air sampling was performed, as validated occupational methods require laboratory-based analysis and were not feasible in the acute phase. Accordingly, airborne concentrations could not be measured, and exposure time was used as a pragmatic surrogate for dose. While hydrazine release was confirmed, potential co-exposures typical of aircraft incidents (e.g., fuels, carbon fiber, polymers) cannot be excluded. Although no fire occurred at the crash site, we acknowledge that the high temperatures generated by the impact may have caused partial thermal decomposition of hydrazine, producing ammonia, which could have contributed to some of the observed symptoms.

The study included only individuals who sought medical care, introducing potential selection bias. All patients were contacted at 7 days, and none reported ongoing symptoms; however, longer-term outcomes were not assessed. Confidence intervals are wide due to the small sample size, and multiple comparisons were conducted in an exploratory context.

Future studies should incorporate quantitative environmental monitoring, standardized biomarker assessments, and extended longitudinal follow-up to validate these findings and better characterize dose–response relationships and potential delayed effects.

## Conclusions

This study provides one of the most extensive civilian datasets on acute hydrazine exposure in an open-field setting. Symptom development was strongly associated with exposure duration, with no patient developing symptoms after ≤ 5 min, while longer exposures—particularly with combined inhalation and physical contact—were linked to clinically significant manifestations such as acute hypoxemic respiratory failure. All patients recovered rapidly with supportive care, underscoring the importance of prompt decontamination and early clinical assessment.

These findings should be interpreted in light of the single-center, retrospective design and the absence of environmental concentration measurements. Exposure time was used as a pragmatic surrogate for dose, highlighting the need for future studies incorporating quantitative environmental monitoring, biomarker assessments, and larger data sets to refine dose–response relationships.

Operationally, these observations support immediate evacuation, timely decontamination, and short-term monitoring of individuals with exposures longer than 5 min. Ensuring appropriate personal protective equipment and strengthening toxicology education and training for first responders remain critical. More broadly, this evidence can inform disaster preparedness and response planning, contributing to both civilian and military readiness for future hydrazine-related incidents.

## Supplementary Information

Below is the link to the electronic supplementary material.

Supplementary Material 1

Supplementary Material 2

## Data Availability

The datasets used and analyzed during the current study are available from the corresponding author upon reasonable request.

## Abbreviations

AUC: Area Under the Curve
CBRN: Chemical, Biological, Radiological, and Nuclear
CI: Confidence Interval
CNS: Central Nervous System
ECG: Electrocardiogram
EPU: Emergency Power Unit
ESICM: European Society of Intensive Care Medicine
GCS: Glasgow Coma Scale
HPLC: High-Performance Liquid Chromatography
IQR: Interquartile Range
OR: Odds Ratio
PaO_2_: Partial Pressure of Oxygen (arterial)
ROC: Receiver Operating Characteristic
SaO_2_: Arterial Oxygen Saturation
SD: Standard Deviation
SpO_2_: Peripheral Oxygen Saturation

## References

[1] Agency for Toxic Substances and Disease Registry (ATSDR). Toxicological profile for hydrazines. Atlanta (GA): US Department of Health and Human Services, Page. last reviewed; May 06, 2014 [Accessed 9 August 2025] 2014. Available from: https://www.atsdr.cdc.gov/toxprofiles/tp100.pdf

[2] United States Air Force. *Thunderbirds Support Manual.* [Internet]. Arlington (VA): USAF; 2025 May 7 [cited 2025 Aug 12]. Available from: https://www.airforce.com/content/dam/airforce/en/pdf/2025ThunderbirdsSupportManual.pdf

[3] Nguyen HN, Chenoweth JA, Bebarta VS, Albertson TE, Nowadly CD. The toxicity, pathophysiology, and treatment of acute hydrazine propellant exposure: a systematic review. Mil Med. 2021;186(3–4):e319–26. 10.1093/milmed/usaa429. 33175959 10.1093/milmed/usaa429

[4] Superfund Health Risk Technical Support Center National Center for Environmental Assessment Office of Research and Development U.S. Environmental Protection Agency Cincinnati. Provisional Peer-Reviewed Toxicity Values for Hydrazine (CASRN 302-01-2), OH 45268. 10 Sep 2009. [Accessed 12 August 2025] Available from: https://cfpub.epa.gov/ncea/pprtv/documents/Hydrazine.pdf

[5] Nguyen HN, McElyea CWE, Chenoweth JA, Nowadly CD, Varney SM, Wilson BZ, et al. Acute exposure to hydrazine reported to four united States regional poison centers: reconsidering a paradigm. Clin Toxicol. 2024;62(5):322–8. 10.1080/15563650.2024.2350601. 10.1080/15563650.2024.2350601 38813683

[6] Sotaniemi E, Hirvonen J, Isomaki H, Takkunen J, Kaila J. Hydrazine toxicity in the human. Report of a fatal case. Ann Clin Res. 1971;3(1):30–3. 5546553

[7] Arch, Chemicals, Inc. *H-70 Propellant: Technical Data Sheet.* [Internet]. Westlake (LA): Arch Chemicals; 2007 [cited 2025 Aug 12]. Available from: https://www.arxada.com/content/dam/arxada/CMC/hydrazine/documents/H-70TDS.pdf.coredownload.pdf

[8] Supplemental flight manual HAF. series aircraft F 16 C/D blocks 50 and 52 + T.O. GR1F-16CJ-1. Lockheed Martin Corporation; 2002 change-1 in 15 June 2003. Available from: https://info.publicintelligence.net/HAF-F16.pdf [Accessed 12 August 2025].

[9] Occupational Safety and Health Administration (OSHA). Method 20: Hydrazine. Washington (DC) U.S. Department of Labor, Occupational Safety and Health Administration. 1989. [accessed 12 August 2025]. Available from: https://www.osha.gov/sites/default/files/methods/osha-20.pdf

[10] Nelson LS, Howland MA, Lewin NA, Smith SW, Goldfrank LR, Hoffman RS, editors. Goldfrank’s toxicologic emergencies. 11th ed. New York: McGraw-Hill Education; 2019. p. 1744. Part C, The Clinical Basis of Medical Toxicology; Chap. 126 Chemical Weapons.

[11] National Institute for Occupational Safety and Health (NIOSH). Hydrazine: Method 3503. In: NIOSH Manual of Analytical Methods (NMAM). 4th ed. Cincinnati (OH): U.S. Department of Health and Human Services, Centers for Disease Control and Prevention. 1994 Aug 15 [cited 2025 Aug 12]. Available from: https://www.cdc.gov/niosh/docs/2003-154/pdfs/3503.pdf

[12] Republic of Turkey Ministry of Health. Algorithm for the approach to chemical and biological threats [Internet]. Ankara: Ministry of Health. 2019 [cited 2025 Aug 15]. Available from: https://dosyamerkez.saglik.gov.tr/Eklenti/33280/0/kimyasal-biyolojik-tehditlere-yaklasim-algoritmasipdf.pdf

[13] Grasselli G, Calfee CS, Camporota L, Poole D, Amato MBP, Antonelli M, et al. ESICM guidelines on acute respiratory distress syndrome: definition, phenotyping and respiratory support strategies. Intensive Care Med. 2023;49(7):727–59. 10.1007/s00134-023-07050-7. 37326646 10.1007/s00134-023-07050-7 PMC10354163

[14] Frierson WB. Use of pyridoxine HCl in acute hydrazine and UDMH intoxication. Ind Med Surg. 1965;34:650–1. 14334180

[15] Chen TM, Malli H, Maslove DM, Wang H, Kuschner WG. Toxic inhalational exposures. J Intensive Care Med. 2013;28(6):323–33. 10.1177/0885066611432541. 22232204 10.1177/0885066611432541

[16] Chao KY, Lin YW, Chiang CE, Tseng CW. Respiratory management in smoke inhalation injury. J Burn Care Res. 2019;40(4):507–12. 10.1093/jbcr/irz043 30893426 10.1093/jbcr/irz043

[17] Nelson LS, Howland MA, Lewin NA, Smith SW, Goldfrank LR, Hoffman RS, editors. Goldfrank’s toxicologic emergencies. 11th ed. New York: McGraw-Hill Education; 2019. p. 663. Part C, The Clinical Basis of Medical Toxicology; Chap. 44, Vitamins.

[18] Kirklin JK, Watson M, Bondoc CC, Burke JF. Treatment of hydrazine-induced coma with pyridoxine. N Engl J Med. 1976;294(17):938–9. 10.1056/NEJM197604222941708. 815813 10.1056/NEJM197604222941708

[19] Dhennin C, Vesin L, Feauveaux J. Burns and the toxic effects of a derivative of hydrazine. Burns Incl Therm Inj. 1988;14(2):130–4. 10.1016/0305-4179(88)90218-5. 3292016 10.1016/0305-4179(88)90218-5

