# Supplementary material for: Civilian mass exposure to hydrazine after an F-16 crash: a retrospective descriptive study
Source: BMC Emerg Med. 2025 Oct 22;25:212. doi: 10.1186/s12873-025-01373-y (PMC12542269; doi:10.1186/s12873-025-01373-y)
Supplement: Supplementary file 1 — Supplementary Material 1 [file 12873_2025_1373_MOESM1_ESM.docx]

**Supplementary Table S1. Exploratory ROC analysis of exposure time for predicting any acute symptom**

| Item | Specification |
| --- | --- |
| **Outcome** | Any acute symptom (yes/no) at ED presentation |
| **Predictor** | Exposure time (minutes), continuous |
| **Primary analyses** | Spearman correlations by symptom category; group comparisons (t-test / Mann–Whitney) |
| **Exploratory ROC** | ROC curve using Youden’s J statistic to identify a data-driven threshold |
| **Data-driven threshold** | 5 minutes (exploratory) |
| **Apparent AUC** | 1.00 (dataset-specific) |
| **Bootstrap 95% CI** | 1.00 – 1.00 (1,000 resamples) |
| **Apparent sensitivity / specificity** | 1.00 / 1.00 (optimistic, unvalidated) |
| **Internal validation** | None (no resampling beyond bootstrap CI; no optimism correction applied) |
| **Key caveat** | Exposure time used as a surrogate for dose; quantitative hydrazine concentration unavailable; findings are hypothesis-generating and dataset-specific |

*Note:* ***ROC analysis identified a 5-minute threshold separating symptomatic from asymptomatic individuals. Given the small sample size, midpoint-imputed exposure durations, and lack of quantitative concentration data, this finding should be considered hypothesis-generating and not prescriptive.***
