# Supplementary material for: Civilian mass exposure to hydrazine after an F-16 crash: a retrospective descriptive study
Source: BMC Emerg Med. 2025 Oct 22;25:212. doi: 10.1186/s12873-025-01373-y (PMC12542269; doi:10.1186/s12873-025-01373-y)
Supplement: Supplementary file 2 — Supplementary Material 2 [file 12873_2025_1373_MOESM2_ESM.docx]

## ****Supplementary Table S2. Effect sizes with 95% confidence intervals for primary comparisons****

| Comparison | Groups (n) | Test | p-value | Effect size | 95% CI |
| --- | --- | --- | --- | --- | --- |
| **Respiratory symptoms** | 4 vs 26 | Mann–Whitney U | 0.0017 | r_rb = 0.962 | 0.885 – 1.000 |
| **Dermatologic symptoms** | 8 vs 22 | Mann–Whitney U | 0.093 | r_rb = 0.398 | 0.074 – 0.727 |
| **Ophthalmologic symptoms** | 11 vs 19 | Mann–Whitney U | 0.129 | r_rb = 0.330 | 0.033 – 0.689 |
| **Gastrointestinal symptoms** | 1 vs 29 | Mann–Whitney U | 0.208 | r_rb = 0.759 | 0.517 – 0.931 (exploratory) |
| **Dermatologic by route** | 0/19 vs 8/11 | Fisher’s exact | 2.8×10⁻⁵ | OR = 94.7 | 4.39 – 2041.9 |
| **Respiratory by route** | 0/19 vs 4/11 | Fisher’s exact | 0.012 | OR = 23.4 | 1.12 – 489.5 |
| **Ophthalmologic by route** | 9/19 vs 2/11 | Fisher’s exact | 0.14 | OR = 0.247 | 0.042 – 1.460 |
| **Gastrointestinal by route** | 0/19 vs 1/11 | Fisher’s exact | 0.208 | OR = 5.57 | 0.21 – 149.17 |

*Note: Effect sizes are reported as rank-biserial correlation (r_rb) for Mann–Whitney U tests and odds ratios (OR) for Fisher’s exact tests, with 95% confidence intervals. For comparisons with zero cell counts, the Haldane–Anscombe correction was applied. The gastrointestinal category (n=1) is exploratory due to very small sample size*
